# In Silico Prediction of Transcription Factor Collaborations Underlying Phenotypic Sexual Dimorphism in Zebrafish (*Danio rerio*)

**DOI:** 10.3390/genes12060873

**Published:** 2021-06-07

**Authors:** Shahrbanou Hosseini, Armin Otto Schmitt, Jens Tetens, Bertram Brenig, Henner Simianer, Ahmad Reza Sharifi, Mehmet Gültas

**Affiliations:** 1 Molecular Biology of Livestock and Molecular Diagnostics Group, Department of Animal Sciences, University of Göttingen, 37077 Göttingen, Germany; bbrenig@gwdg.de; 2 Functional Breeding Group, Department of Animal Sciences, University of Göttingen, 37077 Göttingen, Germany; jens.tetens@uni-goettingen.de; 3 Institute of Veterinary Medicine, University of Göttingen, 37077 Göttingen, Germany; 4 Center for Integrated Breeding Research (CiBreed), University of Göttingen, 37075 Göttingen, Germany; armin.schmitt@uni-goettingen.de (A.O.S.); hsimian@gwdg.de (H.S.); rsharif@gwdg.de (A.R.S.); gueltas@informatik.uni-goettingen.de (M.G.); 5 Breeding Informatics Group, Department of Animal Sciences, University of Göttingen, 37075 Göttingen, Germany; 6 Animal Breeding and Genetics Group, Department of Animal Sciences, University of Göttingen, 37075 Göttingen, Germany; 7 Faculty of Agriculture, South Westphalia University of Applied Sciences, 59494 Soest, Germany

**Keywords:** sexual dimorphism, zebrafish, transcription factor, PC-TraFF, sex gene, colour gene

## Abstract

The transcriptional regulation of gene expression in higher organisms is essential for different cellular and biological processes. These processes are controlled by transcription factors and their combinatorial interplay, which are crucial for complex genetic programs and transcriptional machinery. The regulation of sex-biased gene expression plays a major role in phenotypic sexual dimorphism in many species, causing dimorphic gene expression patterns between two different sexes. The role of transcription factor (TF) in gene regulatory mechanisms so far has not been studied for sex determination and sex-associated colour patterning in zebrafish with respect to phenotypic sexual dimorphism. To address this open biological issue, we applied bioinformatics approaches for identifying the predicted TF pairs based on their binding sites for sex and colour genes in zebrafish. In this study, we identified 25 (e.g., STAT6-GATA4; JUN-GATA4; SOX9-JUN) and 14 (e.g., IRF-STAT6; SOX9-JUN; STAT6-GATA4) potentially cooperating TFs based on their binding patterns in promoter regions for sex determination and colour pattern genes in zebrafish, respectively. The comparison between identified TFs for sex and colour genes revealed several predicted TF pairs (e.g., STAT6-GATA4; JUN-SOX9) are common for both phenotypes, which may play a pivotal role in phenotypic sexual dimorphism in zebrafish.

## 1. Introduction

Transcription factors (TFs) are a large class of DNA-binding proteins that play a central role in controlling the rate of transcription. They bind to specific DNA sequence motifs, *cis*-regulatory elements, in promoter and/or enhancer/silencer regions to regulate gene expression [1,2]. TFs and their cooperation play major roles for the regulation of gene expression (up- or down-regulation) in appropriate time windows and cell types in living organisms [1]. In eukaryotes, it is well established that groups of TFs act together in a combinatorial and coordinated fashion to direct complex body planning, cell division, cell growth, and cell death throughout the life in response to internal and external signals [3,4,5]. 

Sex determination is a complex biological process that directs the undifferentiated bipotent gonad to develop into either a testis or an ovary [6]. This mechanism leads to a stable sexual fate in most animal classes, whereas in some animal classes such as teleost fish species, sex can be reversed during the critical developmental period [7]. Nevertheless, sex differentiation of the gonad is not irreversible even in species with a stable sex determination mechanism and with no sex change in the course of their life, such as in mammals [8]. Indeed, the sexually differentiated gonad requires constant maintenance by the expression of sex genes, which are regulated by sex-specific TFs [8,9]. In mammals, sex is determined by the XX/XY sex chromosomal system, where sex differentiation is initiated by the sex-determining factor encoded by the Y chromosome (SRY in humans and Sry in mice) and mediated by sex hormones [10,11,12,13]. *Sry* is a testis-determining gene in mammals that regulates gene networks through *Sox9* expression to initiate the testis differentiation. The alternative genetic networks, including ovarian-determining genes such as *RSPO1*, *Wnt4/β-catenin*, and *Foxl2* regulate female gonadal development [14]. In this process, gonadal hormones contribute to sex differences by influencing gene expression. Sex differences in adulthood result from differences in gene expression patterns and tissue-specific regulation of sexually dimorphic gene expressions [13]. A number of TFs such as SRY, SOX9, and NR5A1 are known to regulate the sex-determining pathway in mammals resulting in sexual differentiation [15].

In zebrafish (*Danio rerio*), a widely used model animal in scientific research, sex determination in wild populations is regulated by the ZZ/ZW genetic system [16], whereas domesticated strains lack any chromosomal sex determination and sex is determined based on a polygenic system [17,18]. In our previous studies, we have detected a polygenic sex-determination system in domesticated zebrafish strains [19,20]. In wild populations, the W chromosome is indispensable for female primary sex determination, but it is not absolutely associated with its sexual development, as a fraction of ZW individuals develop into males [8,16]. The same is true in medaka fish, which have a XX/XY sex-determination system [21,22]. This evidence shows that although a genetic sex determination mechanism can strongly influence the sexual fate of an individual and thereby the maintenance of a balanced sex ratio under normal conditions, it can be influenced or overridden under certain environmental conditions. It is therefore crucial to understand the underlying mechanism of sex determination and gonad differentiation that enables animals to maintain a stable sexual fate, or to adapt flexibly to the mechanism of sex reversal if this is beneficial in evolution. In zebrafish, many TFs have been reported to play an important role in regulating sex determination and gonad differentiation in particular Dmrt1, Figla, and Nr0b1 [23,24,25]. Dmrt1 is essential for regulating the expression of *amh* and *foxl2* genes, which are necessary for male and female sexual development, respectively. Figla, an oocyte-specific transcription factor (TF), is expressed in the zebrafish ovary during early oocyte development and regulates the expression of zona pellucida subfamily genes for oogenesis such as *zp2* and *zp3* [24]. In the early stage of bipotential gonad development in the zebrafish embryo, Nr0b1 regulates sex determination by influencing germ cell proliferation and expression of pro-female genes such as *cyp19a1a* to promote female development and its expression persists into adulthood [26]. 

Various TFs also regulate pigment cells and colour gene expression in fish species such as Pax3, Sox10, and Mitf [27,28,29,30,31]. Colour patterns in many animal species are a prominent feature that plays an important role in communication, adaptation, mimicry, shoaling, camouflage, and mate choice [32,33]. Pigment patterns play a central role in sexual selection and are strongly conserved during evolution. Sexual attraction using colour patterning and body ornaments in animals is known to be an important signal between individuals influencing mating success [34]. Body colouration can therefore be considered as a secondary sexual trait in sexually dichromatic animal species affecting reproductive success, in which case sexual selection is more pronounced on males than females [35,36]. Mating preferences using body pigmentation have been observed in some fish species such as *Chrysiptera cyanea* and *Gasterosteus aculeatus* [37,38,39]. 

Zebrafish use visual information such as body shape, stripe patterns and colouration for reproductive behaviour [40,41,42,43,44]. There is a sex-associated phenotypic difference in colouration between the two sexes of zebrafish; males show a slightly more intense yellow colouration than females, which may play an important role in sexual attraction [44,45,46,47]. Previous studies indicate that female zebrafish are able to recognize males based on their yellow colouration, particularly during courtship and spawning [48,49]. In zebrafish, Mitf is known as a master TF regulator in melanophore development, governing melanocyte development, including specification, growth, survival, and differentiation [50]. However, in the signaling network of melanophore development, Mitf acts in a co-regulatory fashion with Sox10, Pax3, Lef1, and Creb [50,51,52]. The existence of Sox10 binding sites in the promoter of *mitfa* is necessary to regulate the development of melanophores, which shows that Sox10 directly regulates *mitfa* gene expression [51]. In addition, Sox10 also influences Pax3, suggesting that the cooperation between Pax3-Sox10-Mitf may cooperate and regulate the expression of pigment development in zebrafish [29]. Pax3 is also required for development and specification of xanthophores (yellow pigmentation) in zebrafish [29].

The majority of sexually dimorphic phenotypes arise from differences in sex-biased gene expression patterns present in both sexes [53,54]. This is well documented in a range of species, including *Drosophila* [55,56,57], birds [58,59], mammals [60,61], and fish species such as guppy and zebrafish [53,54,62]. In general, sex-biased gene expression may evolve differently under selection pressures that affect the sexual phenotypes [53,63]. In our recent study on phenotypic sex classification of zebrafish based on colouration using machine learning methods, we detected a higher pigmentation intensity in male zebrafish than in females [47]. Our further investigation of the genetic mechanisms underlying phenotypic plasticity in zebrafish resulted in a series of pathways containing sex and colour genes, which may play a pivotal role in the regulation of phenotypic sexual dimorphism [19]. However, knowledge of sex-related colour pattern variation in two different sexes of zebrafish and its underlying genetic regulation is still limited. 

In the present study, we questioned how the phenotypic differences in pigmentation of male and female zebrafish are regulated. Understanding the regulatory mechanisms of sex and sex-associated colour development can provide new insights into their complex transcriptional machinery that might lead to a better understanding of the underlying mechanism of sexual selection. Hence, the main objective of this study was to investigate the TF pairs, which bind to the regulatory regions of sex and colour genes in a cooperative manner. These predicted cooperative binding of TFs could provide essential information to better understand phenotypic sexual dimorphism in zebrafish, which is important for sexual selection and sexual attraction for mating success. 

## 2. Materials and Methods

### 2.1. Candidate Sex and Colour Genes

**Figure 1:**
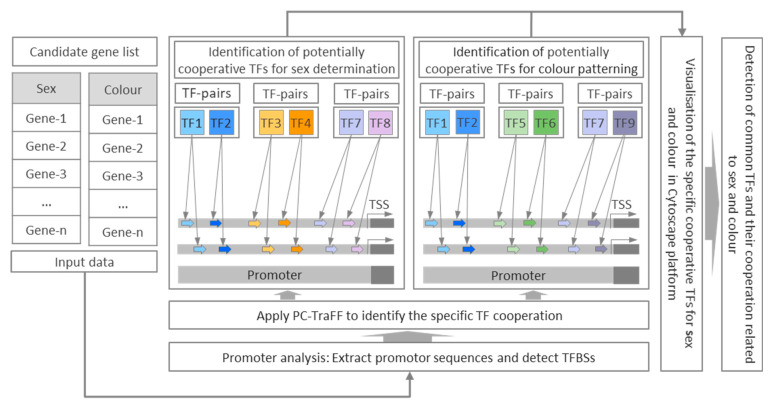
Workflow of analysis used in this study for identification of TF pairs based on their predicted binding sites for sex determination and colour pattern genes in zebrafish with respect to phenotypic divergence. The potential schematic cooperation between TF pairs illustrating that TF1–TF2 pairs are common to both phenotypes (sex and colour), TF3–TF4 pairs are specific to sex determination, TF5–TF6 pairs are specific to colour pattern, TF7–TF8 and TF7–TF9 pairs switch their partners in different phenotypes, where TF7 is a common single TF for both phenotypes. TF: transcription factor, TSS: transcription start site, TFBSs: transcription factor binding sites.

To investigate the predicted cooperative binding patterns of TF pairs underlying phenotypic sexual dimorphism in zebrafish, we used a series of sex- and colour-related genes to identify TFs and their potential complex interplay. By analyzing the predicted promoter regions of a set of genes associated with sex determination and colour pattern in zebrafish, we aim to detect the specific and common TF pairs for both phenotypes (sex and colour). For this purpose, in this study, the expressed sex-related gene sets in zebrafish gonads for sex determination and colour-related gene sets in zebrafish caudal fins for colour pattern were analyzed using a bioinformatics approach from transcriptomic datasets of our previous study [19]. To this end, a total of 73 genes for sex determination and 187 genes for colour pattern were used as candidate genes in this study (Supplementary Materials, Table S1). The workflow of our analysis method in this research work is shown in Figure 1. 

To generate the transcriptomic datasets in our previous study [19], we used the Singapore strain of zebrafish, a laboratory strain that has been maintained in our laboratory facilities since 1990. To quantify the level of expression of sex- and colour-related genes and their association, we generated two lists of candidate genes: (1) a list of genes associated with sex determination; and (2) a list of genes associated with colour pattern addressed in various literature and the NCBI gene database for zebrafish. The list of candidate genes for sex and colour and validation of their expression was reported in Hosseini et al. [19]. The candidate genes were then mapped to the transcriptome profiles (RNA-seq) of 48 adult zebrafish gonads and caudal fins to identify the expression levels of sex and colour genes in both tissues and their associations with respect to sex-associated phenotypic differences in zebrafish colouration. The expression level of most candidate genes was identified in the gonads and caudal fins, while some of them were not expressed in the adult stage of this studied zebrafish strain. Therefore, we used the expressed sex and colour genes of this strain to further investigate their underlying transcriptional regulation by identifying TFs and their potential combinatorial interplay to express different phenotypes regarding phenotypic sexual dimorphism in zebrafish.

### 2.2. Identification of Potentially Cooperative Transcription Factors

In the present study, we applied bioinformatics approaches, namely PC-TraFF [64] and its extension PC-TraFF^+^ [65], for the identification of potentially collaborating TF pairs for sex and colour genes in the zebrafish genome. The main PC-TraFF algorithm is based on information theory and uses the pointwise mutual information (PMI) metric in order to measure the potential cooperation level of TFs regarding their TF binding sites (TFBSs) co-occurrences in the promoters of the genes under study. For this purpose, the algorithm requires the following input parameters:

Promoter sequences: For each gene, we extracted the promoter areas ranging from −500 bp to +100 bp relative to its transcription start site (TSS) using the UCSC genome browser [66]. As mentioned in previous studies [64,67,68,69,70,71,72], the selection of the promoter regions in our analysis is essential to eliminate the overestimation of some TFBSs that often arise from redundancy between sequences and to reduce the potential noisy effect of imprecise prediction of TSS positions. Especially, for the elimination of the highly redundant and correlated promoter sequences, which can arise from gene duplication or TSS annotations of genes, we filtered them as suggested in PC-TraFF [64,65]. Consequently, we considered only non-overlapping promoter sequences in this study, which are not overlapped with other promoters.

Position weight matrix library: For the identification of putative TFBSs in the promoter sequences, we applied the MATCH program [73] together with the default non-redundant vertebrate position weight matrix (PWM) library from the TRANSFAC database [74] included in the PC-TraFF algorithm.

Pre-defined distances: The PC-TraFF algorithm scans the promoter sequences for the co-occurrence of TFBSs to form TF dimers based on their TFBS pairs if the distance preferences between corresponding TFBSs satisfies the user-specified distances. In this study, the recommended distance values were used: minimum distance ≥ 5 bp and maximum distance ≤ 20 bp. 

Significant TF cooperation: For each TF pair of interest, ${\mathrm{TF}}_{a}$ and ${\mathrm{TF}}_{b\;}$, the PC-TraFF algorithm calculates their cooperation using the PMI metric and provides a $\mathrm{PMI}\left({\mathrm{TF}}_{a};{\mathrm{TF}}_{b}\right)$-value based on their TFBS co-occurrence frequencies in the promoters. However, it has been shown that the calculation of PMI values by PC-TraFF^+^ could be influenced by various properties of promoter sequences like their dinucleotide CA- and GC-content, GC skew [68,69], or the ratio and order of the constituent monomers (mononucleotide composition). Consequently, different types of obstacles could lead to a certain level of background co-occurrences between TFBSs and could then affect the PMI values.

In order to overcome this problem in our analysis, we followed the PC-TraFF^+^ workflow and estimated the level of their background cooperation $\left[\mathrm{AVG}\;\left(\mathrm{PMI}\left({\mathrm{TF}}_{a};{\mathrm{TF}}_{b}\right)\right)\right]$ based on shuffled promoter sequences for each ${\mathrm{TF}}_{a}-{\mathrm{TF}}_{b}\;$pair. Thereafter, the AVG(PMI)-values are subtracted from the initial PMI values:

$${\mathrm{PMI}}^{\mathrm{cor}}\left({\mathrm{TF}}_{a};{\mathrm{TF}}_{b}\right)=\mathrm{PMI}\left({\mathrm{TF}}_{a};{\mathrm{TF}}_{b}\right)-\left[\left(1+\alpha \right)\times \mathrm{AVG}\left(\mathrm{PMI}\left({\mathrm{TF}}_{a};{\mathrm{TF}}_{b}\right)\right)\right],$$

where α∈ [−1,+1] refers to a preassigned scaling factor to control the influence of the background level. In our analysis, we used the recommended α = 0.2. It is important to note that, the underlying methodologies of both PC-TraFF algorithms calculate the $\mathrm{PMI}/{\mathrm{PMI}}^{\mathrm{cor}}$ values by summing up the frequencies of TFBS co-occurrences in the promoter’s sequences. This process leads to the identification of only those TF-pairs with strong $\mathrm{PMI}/{\mathrm{PMI}}^{\mathrm{cor}}$ values as significant, which regularly binds to a high amount (almost all) of the promoters in a cooperative manner. 

Finally, ${\mathrm{PMI}}^{\mathrm{cor}}\left({\mathrm{TF}}_{a};{\mathrm{TF}}_{b}\right)$-values are transformed into z-scores and the cooperation between ${\mathrm{TF}}_{a}\;$and ${\mathrm{TF}}_{b}$ is defined to be (PC-TraFF) significant if they have a z-score ≥ 3, as recommended in both PC-TraFF and PC-TraFF^+^ studies [64,65].

## 3. Results and Discussion

**Figure 2:**
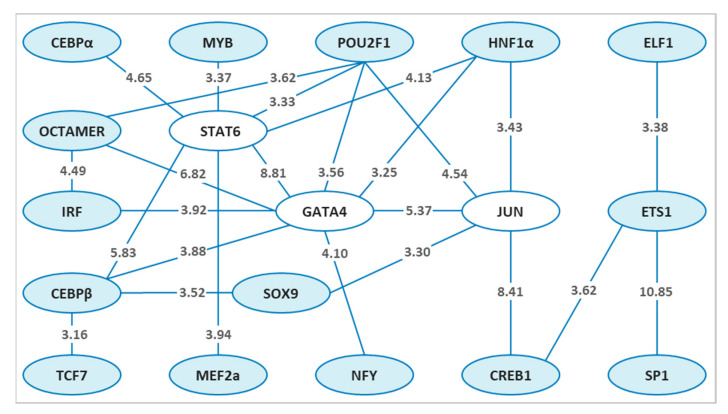
The significant specific transcription factor pairs for sex determination genes in zebrafish. Nodes represent the identified transcription factors for sex in blue or white, and edges represent their potential cooperation. The top three highly connected transcription factors (hubs) in the network are shown in white. The z-score for each transcription factor pair is illustrated on the connecting line.

**Figure 3:**
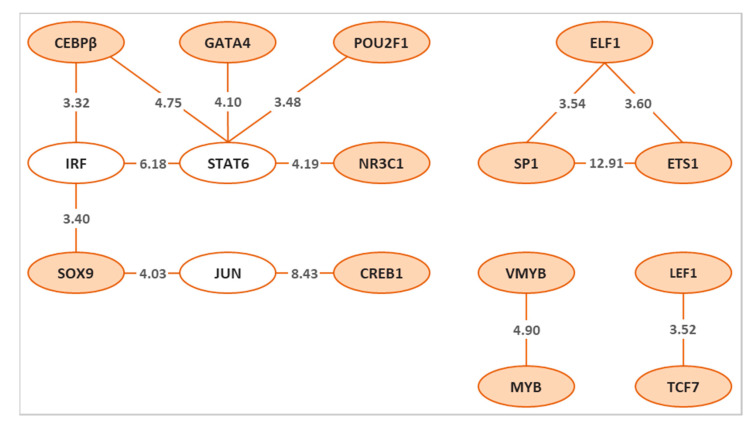
The significant specific transcription factor pairs for colour pattern genes in zebrafish. Nodes represent the identified transcription factors for colour in orange or white, and edges represent their potential cooperation. The top three highly connected transcription factors (hubs) in the network are shown in white. The z-score for each transcription factor pair is illustrated on the connecting line.

**Figure 4:**
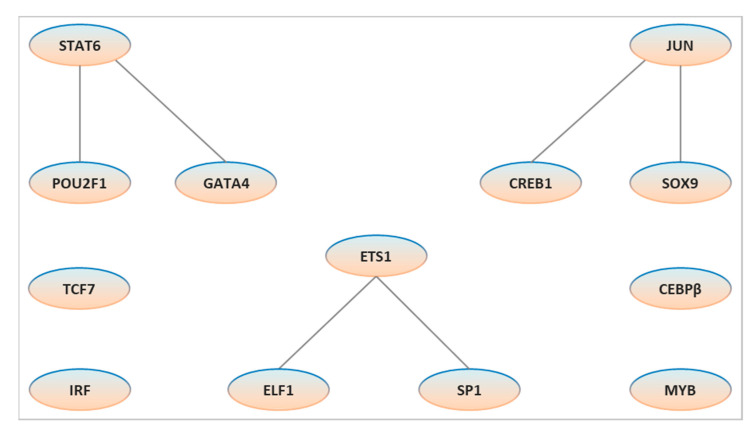
The significant common transcription factor pairs and their potential cooperation network for sex determination and colour pattern in zebrafish. Nodes represent the identified transcription factors for both phenotypes (sex and colour) and edges represent their potential cooperation.

Applying both PC-TraFF algorithms [64,65] to the promoters of previously defined candidate sex determination and colour pattern genes of zebrafish [19] resulted in the identification of 25 and 14 specific potential TF pairs for sex determination and colour pattern, respectively. To gain more insights into the cooperation of these TF pairs for each phenotype, we created a network for sex determination (Figure 2) and colour pattern (Figure 3) genes, which were then compared to identify the common TF pairs for both phenotypes (Figure 4). Interestingly, our findings indicated that although several single TFs overlap in both sex and colour networks, they switch their partners to precisely orchestrate the specific biological processes, which may play an important role in regulating sex and sex-associated colour patterns in zebrafish.

### 3.1. Potentially Cooperative TF Pairs Directing Sex Determination in Zebrafish

Our analysis for candidate sex determination genes in zebrafish demonstrated a pronounced association between potentially cooperative TF pairs in the transcription network for sex determination genes (Figure 2). The z-scores for all significant potentially collaborating TF pairs for sex determination are illustrated on the connecting lines in Figure 2. 

Mainly focusing on the top three highly potentially collaborating TF pairs (hubs) for sex determination genes in the network (Figure 2), we considered GATA4, STAT6 and JUN. In zebrafish, the Gata-family members are well-known TFs that play an important role in the development of many organs, including the intestine, liver, pancreas, swim bladder, and reproductive organs. Zebrafish possess six vertebrate Gata factors (Gata1–6), which are highly conserved at the level of expression pattern and function [75,76]. In zebrafish, the binding sites for GATA4, steroidogenic factor 1 (SF1), and Wilms’ tumor 1 (WT1) are identified on the promoter region of the ovarian-specific gene c*yp19a1a* [77]. 

The *cyp191a* gene encodes the P450 aromatase protein, the key enzyme responsible for the conversion of androgens into estrogens, where estrogens play a crucial role in the anatomical, functional and behavioural characteristics of sexually dimorphic development [8,77]. Estrogens are the key reproductive hormones in females and are involved in female secondary sexual characteristics [8].

In zebrafish, two paralogs of *cyp19a1* were identified, in which *cyp19a1a* is expressed in the ovary and *cype19a1b* is expressed in the brain [77]. The *cyp19a1a* gene, a key sex gene that regulates female sexual development in zebrafish, is expressed in the granulosa cells of the follicle and is a downstream target of Bmp15 [8]. The Bmp15 protein is required for promoting c*yp19a1a* expression in ovaries and maintenance of the adult female sexual phenotype in zebrafish by acting on the somatic gonadal cells of the oocyte follicle [8]. This finding revealed that *cyp19a1a* is necessary for the stabilization of female sexual development and its absence leads to the initiation of testicular development [8]. Zebrafish produce early-stage oocytes during the juvenile ovary stage (10–25 days post-fertilization), where the somatic gonadal cells express a combination of several sex-associated genes to develop either ovary or testis [78,79]. Animals will become male by apoptosis of oocytes in the juvenile ovary, whereas the oocytes in females will continue to mature [80]. Apoptosis that is required for testis differentiation is activated by upregulation of the p53 signaling pathway [81], while activation of the canonical Wnt and NF–κB signaling pathways is required for ovarian differentiation [82,83]. Studies on sex determination and differentiation in zebrafish have shown that besides the direct correlation between germ cell numbers and the sexual development of female zebrafish [84,85], the oocytes signal for female-specific gene expression in the somatic gonad is also required for the development of the ovary and for continuously maintaining the sexual phenotype of adult females [8]. The transcription of *cyp19a1a* via GATA4 TF, as reported previously [77], may play an important role in the sexual fate of zebrafish females in the process of sex determination and gonad differentiation. In this study, we identified the predicted TF pairs along with GATA4 such as STAT6 and JUN for sex determination genes in zebrafish. However, a further experiment is needed to validate our finding of transcriptional activity of these TF pairs in regulating the expression of the *cyp19a1a* gene in zebrafish. 

*Amh* and *sox9a* are two Sertoli cell-expressed genes in the testes of adult zebrafish, in which their expression showed different transcriptional dependencies on Dmrt1 in the sex determination signaling pathway [23]. The sex differentiation gene regulatory network in zebrafish revealed that *dmrt1* is either downstream of or parallel to *sox9a* [23]. However, *amh* is a downstream target of *dmrt1*, and its activation inhibits the expression of *cyp19a1a* leading to testicular development. Indeed, *amh* and *cyp19a1a* act reciprocally in the zebrafish gonad; males with high levels of *amh* expression show lower expression of *cyp19a1a* and vice versa in females [23,86]. The transcriptional regulation of *amh* in zebrafish is not well understood at the present time [87]. However, the promoter sequence of the *amh* gene in zebrafish contains several putative binding sites for the same TFs as in mammals, such as GATA4, WT1, SF1, and SOX9 [79,86]. Further studies are needed to investigate whether these TFs are active in the promoter of the *amh* gene in zebrafish. 

STAT6, the second hub in our sex determination network (Figure 2), is a member of the Signal Transducer and Activator of Transcription (STAT) family of proteins. Activation and binding of the STAT6 to DNA motifs alone is usually not sufficient to start the transcription of a STAT6 target gene. Instead, the cooperation of STAT6 with other TFs or a set of transcriptional co-regulatory proteins can coordinate signals to initiate the transcription of a target gene [88]. In this regard, two important co-factors interacting with STAT6 and contributing to the specificity of the STAT6 transcriptional regulation of a STAT6 target gene are CREB and p300. Further cooperative TFs that are close to the STAT6 motifs and interact synergistically or antagonistically with STAT6 to control the activation of a target gene are the family of CEBP, AP1 and MYB TFs [88]. In accordance with these findings, our analysis identified the predicted binding sites for STAT6 with both CEBPα and CEBPβ, and STAT6 with MYB for sex determination genes in zebrafish. However, this predicted potential cooperation was not detected between STAT6 and the AP1 family of TFs in our analysis, as shown in Figure 2.

The function of STAT6 in the transcriptional activation of the *HSD3B1* and *HSD3B2* gene promoters encoding 3β-HSD type 1 and 3β-HSD type II enzymes, which catalyze the sexual steroid hormone, was detected in human reproductive organs [89,90,91]. It has been found that the *HSD3B2* gene is predominantly expressed in the adrenal gland, ovary, and testicular Leydig cells [90]. A later study on the mediators of the JAK–STAT signaling pathway in human reproduction revealed that the STAT family proteins are present in sperm cells, of which STAT6 was detected in sperm heads [92]. The two mammalian steroidogenic genes *HSD3B1* and *HSD3B2* are present in zebrafish, but they differ in their expression pattern and physiological functions [93]. It has been reported that the transcriptional activation of the *hsd3b* gene in zebrafish is regulated by TF encoded by the *nr5a1a* gene [93]. The NR5A1 is a member of the zinc-finger TFs, which controls the expression of steroidogenic and gonadotropin genes, thereby playing a crucial function in gonadal development and sexual differentiation in zebrafish [94]. However, the regulatory transcriptional function of STAT6 for the expression of steroidogenic genes in the reproductive organ of zebrafish remains to be clarified. Here, we report the potential collaboration of STAT6 with other important TFs such as GATA4, CEBPβ, and SOX9 for transcription of sex-determining genes in zebrafish. 

The third hub in our analysis for candidate sex determination genes in zebrafish was JUN in the sex network (Figure 2), which is encoded by *jun* gene and is the central component of the activator protein 1 (AP1) family [95]. The AP1 TF family is composed of proteins belonging to the FOS, JUN, ATF and JDP families. Members of the AP1 family, especially JUN and FOS, are involved in transcriptional regulation of genes required for sexual development. The Jun TF was shown to act in a cooperative manner with other TFs, e.g., JUN and SF1, JUN and NUR77, JUN and GATA4, in the promoter of the *STAR* gene in the mouse and human genome [96,97]. The functional interplay between these TFs is required for cell-specific expression and hormonal regulation of the *STAR* gene in steroidogenic cells of the testis [97]. The STAR protein transports cholesterol through the mitochondrial membrane to initiate the steroidogenesis and produce testosterone in testicular Leydig cells of males. Testosterone is a male sex hormone, which plays a key role in the normal development of male reproduction and promotes male secondary sexual characteristics [98,99]. In the ovary, the *STAR* gene is expressed during various ovulatory developmental phases [100,101].

The interplay between different TFs such as CREB-CREM and FOS-JUN-CEBPβ can regulate the expression of the *STAR* gene [102,103,104]. The JUN and FOS TFs play a critical role in regulating steroidogenesis in Leydig cells of males [105,106]. In addition to Leydig cells, the transcriptional cooperation between CEBPβ and GATA4 in granulosa cells of porcine and rat has also been reported [107,108]. Furthermore, the cooperation between AP1 elements and GATA factors in the promoter of other steroidogenic genes such as *Cyp11a1* in mice and *HSD3B2* in humans has also been proven [109,110]. 

In zebrafish, the *star* gene is expressed in steroidogenic tissues as in mammals, and its transcripts were detected in the ovary, testis, kidney and head [111]. In the ovary, the expression pattern of *star* was observed to differ between stages of the follicle’s development in sexually mature animals [112]. Its expression remained constant during primary growth through vitellogenic follicles but decreased significantly during maturation [112]. This result implies that the *star* gene is expressed differentially over time at different stages of ovarian follicular development in zebrafish, as they are asynchronous spawners bearing oocytes in various stages of the development [112]. 

In a previous study, a high level of *star* expression was observed in the testis of zebrafish [111]. This indicates a pivotal function of the *star* gene in the steroidogenesis process of reproduction and the sexual development of both male and female zebrafish. In our analysis, we have also identified the potential cooperation between JUN-CREB1, JUN-GATA4, GATA4-CEBPβ TF pairs for sex determination genes in zebrafish, which may play a role in regulating the *star* gene (Figure 2). However, further research is needed to confirm this assumption. Interestingly, we found a further potential cooperation between JUN-SOX9, GATA4-CEBPβ-SOX9 that may have an important function in regulating of sex-determining genes in zebrafish. In mice, the functional cooperation between JUN and SOX9 regulates the expression of the *Gja1* gene within testicular Sertoli cells [113]. The JUN TF cooperates synergistically with SOX8 and SOX9 in the promoter of the mouse *Gja1* gene and activates its expression in Sertoli cells [113]. In this study, the potential cooperation between JUN and some reported TFs in other species such as JUN-SF1, JUN-NUR77, FOS-JUN, and JUN-CEBPβ was not identified in the potential TF cooperation network for sex determination in zebrafish (Figure 2). Our understanding of the precise function of JUN family in a cooperative manner with other TFs to regulate the expression of sex-related genes in zebrafish is still far from being complete and further investigation may allow us to explain its fundamental role in the reproduction process of this animal.

### 3.2. Potentially Cooperative TF Pairs Directing Colour Patterning in Zebrafish

Our analysis of predicted TF pairs based on their cooperative binding sites for colour pattern genes in zebrafish revealed 14 TF pairs, which could provide crucial knowledge for phenotypic sexual dimorphism (Figure 3). The z-scores for all significant potentially collaborating TF pairs for colour pattern are illustrated on the connecting lines in Figure 3. 

Focusing on the three top highly connected TF pairs in this network, we identified STAT6, IRF, and JUN as hubs (Figure 3). STAT6 and JUN were interestingly the same hubs in both networks for sex determination and colour pattern genes. The TF cooperative network for colour pattern genes demonstrated that the potential cooperation of STAT6 and JUN with other TF pairs was surprisingly similar to the observed cooperation of these TFs for sex determination genes. The potential cooperation of STAT6-GATA4 and JUN-CREB1-SOX9 was identified for both networks.

The STAT family of proteins plays an important role in mammalian ocular development and maintenance. The expression and activation of STAT6 in the retinal pigment epithelium of mouse have been observed [114]. Abnormalities in retinal pigment epithelium cells can lead to degeneration of the ocular photoreceptors and consequently to blindness. Age-related macular degeneration in human is a disease resulting from a degeneration of retinal pigment epithelium cells that can cause irreversible loss of central vision [115,116,117,118]. Several studies in animal models revealed that the transplantation of retinal pigment epithelium cells could delay this disease, with the greatest progress being reported in the xenotransplantation of these cells from pig to human [117,118,119,120]. Investigation of the effect of human cytokines on porcine ocular cell activation in xenotransplantation research demonstrated that these cells can receive and transmit human interferon and cytokine signals via the JAK–STAT signaling pathway to activate the STAT family of proteins, and can thereby regulate the transcription of their target genes in the eye [118].

Furthermore, the JAK–STAT signaling pathway has an important function in the regulation of melanogenesis in human skin. Abnormal skin pigmentation, hyper- or hypopigmentation, after inflammation is a common symptom in dermatology [121,122]. Skin inflammation induced in response to exogenous or endogenous stimuli can lead to changes in skin pigmentation [123,124,125,126]. In general, activation of MITF leads to an upregulation of the expression of key melanogenesis genes such as *TYR*, *TYRP-1* and *TYRP-2* in melanosomes, the unique organelles located in the cytoplasm of melanocytes, which promote melanogenesis in melanocytes. Several signaling pathways such as PKA, PKC, MAPK, WNT, and IP3/DAG were shown to be involved in melanogenesis [122]. The interleukin IL-4, a cytokine activator of STAT6 in the JAK–STAT signaling pathway, is secreted by T-helper2 (Th2) cells and plays an important role in downregulating the expression of *MITF*, *TYR, TYRP-1* and *TYRP-2* genes, thereby inhibiting melanogenesis in human [121]. The study of Choi and colleagues [118] demonstrated that STAT1, STAT3, and STAT6 are involved in melanin synthesis and expression of melanogenesis-associated genes and in the JAK2-STAT6 signaling pathway in skin disease [121]. Nevertheless, the regulation of melanogenesis by the JAK–STAT signaling pathway is not yet fully understood and needs to be confirmed by further experiments. To the best of our knowledge, there is no information on the role of STAT6 in colour patterning in zebrafish, a question that deserves further research.

The interferon-regulatory factor (IRF) was the second hub in the colour pattern cooperation network in our analysis (Figure 3). The IRF family of TFs has been reported to play a role in pigmentation in human, mouse, and zebrafish [127,128,129]. In these species, IRF4, a member of the IRF family, cooperates with MITF to activate the expression of the gene encoding the TYR enzyme, the pigmentation enzyme required for melanin synthesis. The melanocyte master regulator MITF binds to an enhancer element in intron 4 of the *IRF4* gene and activates its expression. This activation depends on the presence of TF Activator Protein 2 alpha (TFAP2A). The encoded IRF4 protein binds to the proximal region of the *TYR* pigmentation gene promoter to activate its expression cooperatively with MITF [127,129]. In humans, IRF4 is associated with sensitivity of the skin to sun exposure, freckles, blue eyes and brown hair colour. Reduced expression of *IRF4* leads to lighter hair colour in human, while in mice, the reduction in both *Mitf* and *Irf4* is necessary to decrease the pigmentation, indicating species dependency in hair melanocytes [127]. Besides the co-activating function of Irf4 and Mitf in the melanin synthesis in zebrafish [127], Tfap2a protein is involved in melanocyte differentiation [130]. An effector of Tfap2a and Tfap2e activity is required for melanocyte differentiation in the zebrafish embryo, which acts upstream or parallel to Mitfa activity [130]. Furthermore, the cooperation between Tfap2a and Tfap2e promotes the expression of the zebrafish *kita* gene in embryonic melanocytes, which is also one of the important genes for melanocyte differentiation [130]. Hence, evidence shows that IRF4 TF has major effects on phenotype pigmentation in humans, mice, and zebrafish through the MITF master regulator, where *TYP* is a target gene of the IRF4-MITF cooperative activation [127].

The third hub in the network for colour pattern genes was JUN (Figure 3). The dynamic changes in the AP1 family of proteins, in particular the TFs JUN and FOS, following cellular stress stimuli disrupt the signals that define cell function to become apoptosis, survival or senescence due to the differentially regulated target gene expression [60,131]. In a study of human melanoma disease, the AP1 family of TFs, especially JUN, was shown to play an important role in the prevention and/or treatment of melanoma [60]. Zebrafish is an excellent model animal for modeling melanocyte and studying melanoma diseases in human, since a high percentage of the genes associated with human diseases (82%) have a zebrafish ortholog [132]. Despite the differences in skin structure and melanocyte biology among different species, the melanocytes of zebrafish share many characteristics with the melanocytes of humans. Melanocyte development and melanin synthesis in zebrafish are regulated by *mitf* as a master regulator, *kit* and other genes involved in the MAPK signaling pathway as executors of cell fate, and melanin biosynthesis genes such as *tyr, tyrp1*, and *pmel*. 

Furthermore, the function of AP1 subunits, JUN and FOS, in mouse skin physiology and pathology has been reported as important TFs for the cellular switching of genetic programs in response to extracellular signals and their target gene expression [131]. Likewise, the study of Jun in *Xiphophorus* fish melanoma model indicated the role of AP1 components in melanoma disease [133]. Transcriptome analysis of coat colour in sheep revealed the expression of melanosome component genes such as *DCT, MATP, TYR and TYRP1* in the skin of black sheep [134]. This study identified the expression of multiple TF genes regulating skin colour in sheep, including the expression of general TFs such as DLX3, JUN, ATF4, and GATA3 and TFs known to regulate mRNA expression of coat colour genes such as *MITF* and *CREB/ATF bZIP* [134]. The number of melanosomes is associated with skin and hair colour, darker skin pigmentation possessing a higher number of melanosomes than lighter skin. Among the known genes for coat colour, the function of genes in the category related to ‘the component of melanosome and their precursor’ and ‘Eumelanin and pheomelanin’ are melanin synthesis and the switch between eumelanin and pheomelanin in the skin or hair [134,135]. The association between less melanin with lighter phenotype was observed in humans [136], alpacas [137], llamas [138], and horses [139]. This demonstrates that the AP1 family of TFs, especially the JUN family, plays an important role in melanin synthesis and expression of melanogenesis-associated genes.

### 3.3. Identification of Common Cooperative TFs for Sex and Colour Genes in Zebrafish

A comparison of potentially collaborative TF pairs predicted for sex and colour genes in zebrafish revealed the significant common TF pairs for both phenotypes (Figure 4). This figure also shows that the preferential partner choice of several (6 of 25 for sex and 6 of 14 for colour) TFs is identical for both phenotypes, which appears to play a significant role in the association between sex and colouration, resulting in sex-associated phenotypic dimorphism. In this network, we identified IRF, MYB, TCF7 and CEBP as a single TF because they changed their partner in the sex determination and colour pattern specific cooperation networks. In the present study, we extend our previously published results [19,47] on phenotypic sexual dimorphism in zebrafish to gain new insights into its underlying transcriptional regulation for sex and colour genes in the zebrafish genome, which will contribute to a better understanding of phenotypic sexual dimorphism in this species.

Sexual selection in animal species using body ornaments such as body colouration or body size plays a crucial role in mating preference [34]. The secondary sexual characteristics stand for the sexual attractiveness, which influences the reproductive behavior and mating success, leading to sexual dimorphism [35,36]. The sexually dimorphic phenotypes stem from differences in sex-biased gene expression, which may evolve differently under selection pressures [53,54,63]. Several studies demonstrated that zebrafish use visual information for mating success, with female zebrafish attracting intense yellow-coloured partners during courtship and spawning [44,45,48,49]. Furthermore, it has been reported that male zebrafish show a slightly more intense yellow colouration than females [44,46,47]. However, there is limited knowledge about the role of TFs in gene regulatory mechanisms for sex determination and sex-associated colour pattern in zebrafish due to minor sexual dimorphism in the body colour in this animal.

## 4. Conclusions

To address the limited knowledge underlying genetic mechanisms of sex determination and sex-associated colour patterning in zebrafish with respect to phenotypic sexual dimorphism, we investigated the predicted TF pairs based on their cooperative binding sites in the promoters of sex and colour genes in the zebrafish genome. In particular, we focused on preferential partner choice of TFs for sex and colour pattern in our analysis. Interestingly, we found that several predicted TF pairs are common for both phenotypes. These findings provide valuable information on the functional background of the association between sex and colour genes in zebrafish, inducing phenotypic sexual dimorphism, which is important for sexual selection from the evolutionary point of view. Therefore, additional experiments in molecular biology are needed to validate the functions of these TFs to better understand the genetic background directing both phenotypes.

## Data Availability

The datasets supporting the findings of this study are available within the manuscript and its Supplementary Materials. The datasets presented in this study are available from the corresponding author upon reasonable request.

## Supplementary Materials

The following are available online at https://www.mdpi.com/article/10.3390/genes12060873/s1. Table S1: candidate gene list.
